# Case Report: Cutaneous Squamous Cell Carcinoma Arising From the Ulcer of the Lesions of Dupuytren’s Disease on the Palm

**DOI:** 10.3389/fonc.2021.638395

**Published:** 2021-03-25

**Authors:** Qingmiao Sun, Bin Fu, Sheng Li, Hong Fang, Jianjun Qiao

**Affiliations:** ^1^ Department of Dermatology, The First Affiliated Hospital, Zhejiang University School of Medicine, Hangzhou, China; ^2^ Department of Infectious Diseases, Hangzhou Hospital of Traditional Chinese Medicine, Hangzhou, China

**Keywords:** Dupuytren’s disease, squamous cell carcinoma, palm, skin cancer, surgical excision

## Abstract

Dupuytren’s disease is a benign fibromatosis that mainly involves the fascia of the palm and digits. The relationship between Dupuytren’s disease and the evolution of cutaneous squamous cell carcinoma is still unclear. Here we report the case of a 52-year-old female with squamous cell carcinoma arising from the ulcer of the lesions of Dupuytren’s disease on the left palm. To our knowledge, this is the first reported case in the English literature of squamous cell carcinoma on the palm of someone with Dupuytren’s disease.

## Introduction

Dupuytren’s disease (DD) is a connective tissue disorder of the hand characterized by excessive fibrosis of the palmar fascia. DD results in finger contracture and disability (1). It is usually progressive, irreversible, symmetrical, and with a late-onset. The 4th and 5th digits are most often affected. Prevalence rates range from 0.2% to 56% (2) and the exact pathogenesis of DD remains unclear.

Squamous cell carcinoma (SCC) is the most common skin cancer of the hand. However, SCC associated with DD has been rarely reported. Haslik et al. (3) reported a case of SCC in the presence of DD in the language of German. Alberico et al. (4) reported another case of SCC in the presence of Ledderhose disease. Ledderhose disease is considered to share a spectrum with DD and 10% of the patients of DD can be found with Ledderhose disease (5). To our knowledge, this is the first case in the English literature of SCC on the palm of a patients with DD.

## Case Report

Written informed consent was obtained from the patient for the publication of any potentially identifiable images or data included in this article. The report was approved by the ethics committee of the First Affiliated Hospital, Zhejiang University School of Medicine (Approved number: IIT-2020-664).

**Figure 1:**
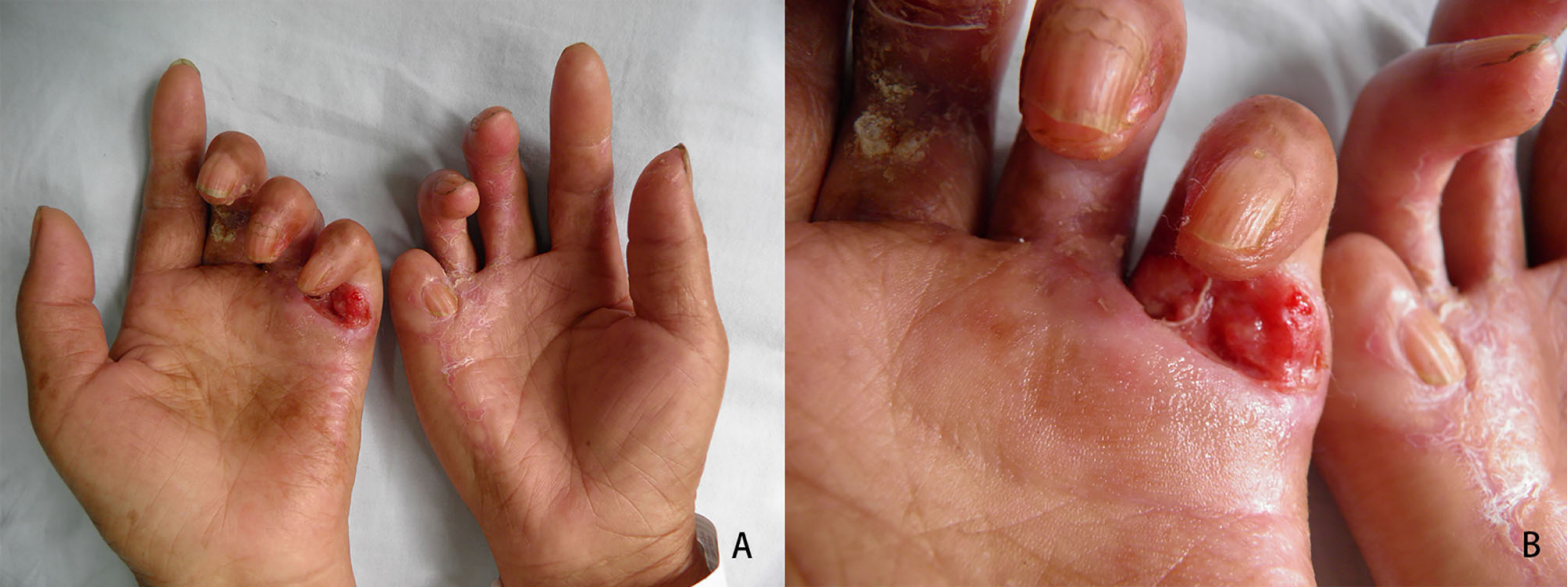
Clinical features of the hand lesions. **(A, B)** Contracture deformity of both hands was evident, and an ulcer was detected on the flexor side of the 5th metacarpophalangeal joint of the left hand.

A 52-year-old female with long standing severe DD affecting 3rd to 5th digits of both hands presented to our department of dermatology with a 1-year history of a painful non-healing slowly enlarging ulcerated nodule on her left palm. The patient did not recall any personal or family history of skin cancer, diabetes mellitus, epilepsy and genetic disease. She also denied history of smoking, alcohol consumption, heavy labor, trauma and immunodeficiency. Physical examination showed a 2.0 × 1.0 cm red ulcer on the flexor side of the 5th metacarpophalangeal joint of the left hand (**Figures 1A, B**). There was flexion contracture of the 3rd to 5th fingers of both hands, and the 5th finger of the right hand was fused with the palm.

**Figure 2:**
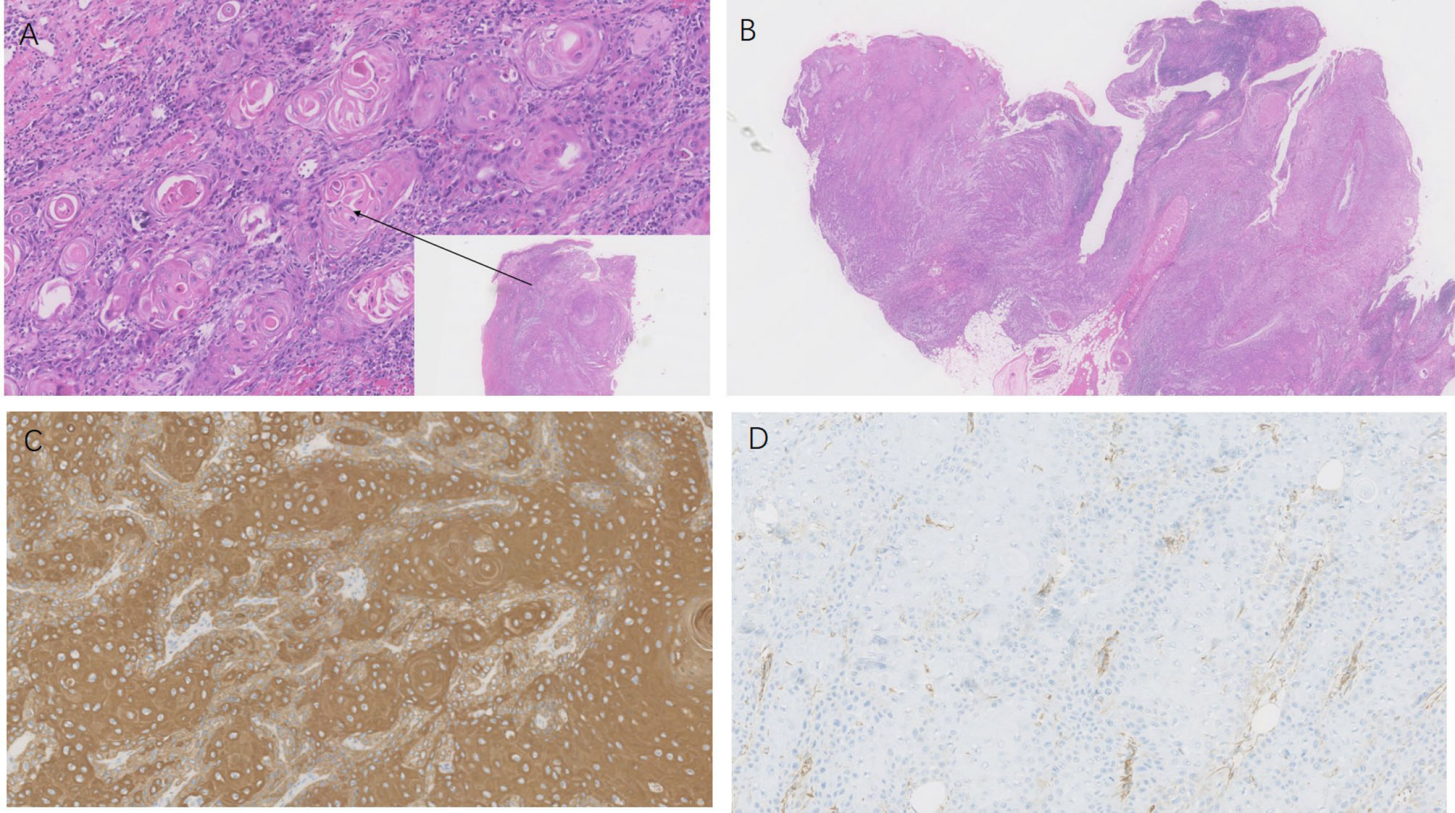
Histopathology of the hand lesion. HE staining of the tissue (panel **A** was from the edge of the ulcer, panel **B** was from the ulcer) revealed atypical squamous cell proliferation and horn pearls in the dermis (H&E×100, ×10). Immunohistochemical staining of the biopsy specimen showed **(C, D)** the squamous cells were positive for pan cytokeratin and negative for vimentin (×100).

No obviously enlarged lymph nodes were evident in the bilateral axilla using ultrasonography. A cutaneous biopsy specimen taken from the edge of the ulcer showed hyperkeratosis of the epidermis, with infiltration of atypical squamatous cells into the dermis with horn pearls. No evidence of fungal or mycobacterium infection was found. Immunohistochemistry showed the atypical squamous cells were positive for pan cytokeratin and negative for vimentin. Well differentiated SCC was diagnosed (**Figure 2**).

**Figure 3:**
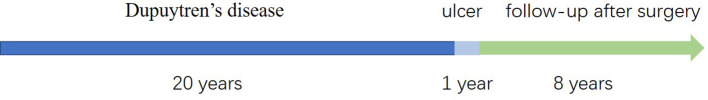
Timeline of the disease’s development.

An axillary lymph node biopsy did not reveal any neoplastic cells. The patient requested an amputation of her left hand at the wrist. The patient has been followed-up for 8 years and has remained free of recurrence of the SCC (**Figure 3**).

## Discussion

DD is considered a benign but debilitating disease due to the decreased range of motion and grip strength. Dysregulation of certain genes may affect the growth characteristics of fibroblasts in palmar aponeurosis, leading to gradual differentiation into myofibroblasts and excessive production of type III collagen (6). Infiltrative growth, proliferation, lack of apoptosis, and a tendency to relapse are the characteristics of DD fibroblasts. One study showed that the incidence of fibrosarcoma and 2malignant fibrous histiocytoma may increase in DD patients (7). However, no correlation has been established between DD and the formation of skin malignancies. Non-melanoma skin cancers (NMSCs) tend to occur less frequently among female DD patients (8). However, it is believed that the prevalence of skin tumors may be underestimated and not adequately documented. A recent retrospective study of 181 DD patients showed that the risk of NMSC in DD patients was twice that of the controls (9). Thus, the relationship between DD and skin cancers remains unclear.

Cutaneous SCC is the most common primary malignancy of the hand. It has an increasing annual incidence, accounting for 20 to 30% of cutaneous malignancies (10, 11) and 58 to 90% of all hand tumors (12, 13). It is almost always located on the dorsum of the hand, with very rare occurrence on the palm (14). Only a few case reports of SCC on the palm have been published (15, 16). The ulcer on the palm of our patient was finally diagnosed with cutaneous SCC. However, it still needs to be differentiated from trauma, pyoderma, fungal or mycobacterium infection.

SCC in the presence of DD is rarely reported as well as in the presence of Ledderhose disease, Garrod knuckle pads, and Peyronie’s disease which share common predisposing factors, comorbidities, pathophysiology, and evolution with DD (5, 9). The two reported cases are mentioned above. To our knowledge, there are no reports of SCC in the presence of Garrod knuckle pads and Peyronie’s disease, and this is the first case in the English literature of SCC on the palm of a patients with DD. It is postulated that chronic inflammation/wound and scarring in DD may be the cause of SCC.

Surgical excision is one of the main treatments for cutaneous SCC. Surgery requires consideration of recurrence rate, function preservation, patient expectation, and potential adverse reaction (17, 18). The current literature on the surgical treatment of cutaneous SCC is conflicting, with no conclusive results on the effectiveness of various prognostic factors and surgical approaches for primary tumors (19). According to the guidelines of care for the management of cutaneous SCC by the American Academy of Dermatology (20), for low-risk primary cutaneous SCC, standard excision is recommended with a margin width of 4 to 6 mm to a depth of the middle layer of subcutaneous adipose tissue by histologic margin assessment. For high-risk primary cutaneous SCC, Mohs micrographic surgery is recommended. Similar to this guidelines, for high risk cutaneous SCC that has not spread to lymph nodes, Mohs surgery or resection with complete circumferential peripheral and deep margin assessment or standard surgical excision is recommended by National Comprehensive Cancer Network (NCCN) guidelines (21). Few studies have focused on the outcomes of SCC of the hand treated by surgical excision, and the use of amputation as a treatment for such SCC is controversial (15). In a retrospective study of 273 patients with SCC of the hand, the death rate from metastasis was 21% with amputation, compared with 7% with radiotherapy alone (22). In the present case, the patient suffered from DD for 20 years with severe hand dysfunction and presented with a 1-year history of non-healing enlarging ulcerated nodule that was confirmed as SCC. According to the risk factors of NCCN guidelines (21), the tumor occurred in an ulcer site on the hand with 20mm in size was of high-risk, and an axillary lymph node biopsy was required. Although the axillary lymph node biopsy was negative, the patient declined to receive Mohs surgery and selected amputation at the left wrist because of the serious functional impairment of her left hand and worried about recurrence.

In summary, DD is a common benign fibromatosis mainly involving the fascia of the palm and digits. However, SCC in the palm accompanying the disease has not been previously described in the English literature. The incidence of SCC in DD is unclear. Further attention should be given to the incidence, and routine dermatological examinations are necessary for such patients.

## Data Availability Statement

The original contributions presented in the study are included in the article/supplementary material. Further inquiries can be directed to the corresponding authors.

## Ethics Statement

Written informed consent was obtained from the patient for the publication of any potentially identifiable images or data included in this article.

## Author Contributions

All authors contributed to the article and approved the submitted version.

## Funding

This work was supported by the Zhejiang Medical and Health Science and Technology Project (2015KYA092).

## Conflict of Interest

The authors declare that the research was conducted in the absence of any commercial or financial relationships that could be construed as a potential conflict of interest.
